# Cells Derived from Concentrated Growth Factor Exhibit a Multilineage Differentiation Capacity

**DOI:** 10.3390/ijms26178646

**Published:** 2025-09-05

**Authors:** Laura Giannotti, Nadia Calabriso, Francesco Spedicato, Andrea Palermo, Benedetta Di Chiara Stanca, Christian Demitri, Maria Antonietta De Sangro, Maria Annunziata Carluccio, Fabrizio Damiano, Luisa Siculella, Eleonora Stanca

**Affiliations:** 1 Department of Experimental Medicine (DiMeS), University of Salento, 73100 Lecce, Italy; laura.giannotti@unisalento.it (L.G.); andrea.palermo@unisalento.it (A.P.); benedetta.dichiara@unisalento.it (B.D.C.S.); christian.demitri@unisalento.it (C.D.); fabrizio.damiano@unisalento.it (F.D.); 2 Institute of Clinical Physiology (IFC), National Research Council (CNR), 73100 Lecce, Italy; nadia.calabriso@cnr.it (N.C.); mariaannunziata.carluccio@cnr.it (M.A.C.); 3 Department of Biological and Environmental Sciences and Technologies (DiSTeBA), University of Salento, 73100 Lecce, Italy; francesco.spedicato@unisalento.it; 4 Institute of Polymers, Composites and Biomaterials, National Research Council (IPCB-CNR), 80125 Naples, Italy; 5 Blood Trasfusion Center, Vito Fazzi Hospital, 73100 Lecce, Italy; madesangro@gmail.com

**Keywords:** concentrated growth factors, stem cells, cell differentiation, circulating stem cells, regenerative medicine, tissue regeneration

## Abstract

Concentrated growth factor (CGF) is an autologous blood-derived product widely used in regenerative medicine due to its high concentration of growth factors and platelets. In this study, the ability of primary stem cells isolated from human CGF to differentiate into adipocytes, endothelial cells, and neuronal-like cells was evaluated in vitro. CGF primary cells (CPCs) were obtained from CGF fragments and characterized after one month in culture. These cells were positive for the surface markers CD105, CD45, CD31, and CD14, and also expressed mRNA levels of the stemness markers Nanog and Oct3/4 comparable to human bone marrow mesenchymal stem cells (BMSCs). Results showed that, following appropriate differentiation protocols, CPCs, similarly to BMSCs, were able to differentiate into adipogenic, endothelial, and neuronal lineages, acquiring specific phenotypic and molecular markers. Adipogenic induction resulted in lipid accumulation and the upregulation of key genes, including PLIN2, FABP4, CD36, and FASN. Under pro-endothelial conditions, the cells exhibited increased expression of endothelial markers, eNOS, VEGFR-2, and CD31. Neuronal induction promoted the expression of β-tubulin III, Nestin, and Neurofilament. Overall, this work highlights the remarkable plasticity of CPCs and supports their potential application in multilineage regenerative therapies.

## 1. Introduction

The use of autologous platelet concentrates (APCs) in regenerative medicine has been steadily increasing due to their ability to promote wound healing and tissue regeneration [1]. Certain blood components, such as peripheral blood mononuclear cells, may contain a small fraction of circulating stem cells or multipotent progenitor cells involved in the inflammatory response and in the tissue repair process following injury [2,3,4]. Under specific microenvironmental conditions, monocytes can also exhibit pluripotent features and the ability to differentiate into various cell types, including epithelial, endothelial, neuronal cells, and hepatocytes [4,5]. The cell populations isolated from APCs, including monocytes, provide a valuable resource for studying cellular responses in regenerative contexts. These cells have demonstrated potential differentiation capabilities into multiple lineages, although they express distinct markers compared with mesenchymal stromal cells typically used in medical applications [6].

APCs are enriched in cytokines, growth factors, and extracellular matrix proteins that play crucial roles in cell proliferation, migration, and differentiation. Recent advances in the preparation and clinical application of platelet derivatives have further expanded their therapeutic potential. Studies have shown that the application of APCs can significantly improve healing outcomes in pathological conditions such as diabetes, alopecia, or nerve injuries [7,8,9,10].

Concentrated growth factor (CGF), a third-generation of APC, contains high levels of growth factors, leukocytes, and a heterogeneous population of cells expressing surface markers typical of hematopoietic stem cells and mesenchymal stromal cells, including endothelial progenitor cells [11,12,13]. Owing to their ability to support the recruitment, proliferation, and maturation of cells involved in tissue regeneration, platelet concentrates are particularly beneficial for poorly vascularized tissues with slow cell turnover and limited extracellular matrix repair capacity [14].

Transforming growth factor β (TGF-β), bone morphogenetic proteins (BMPs), vascular endothelial growth factor (VEGF), and matrix metalloproteinases (MMPs), such as MMP-2 and MMP-9, are present at high concentrations in CGFs and are released gradually and sustainably over time. This property makes CGFs especially well-suited to supporting the later stages of regeneration, in contrast to other APCs that release their growth factors more rapidly [15,16].

We previously characterized CGF from both morphological and functional perspectives. One of the most important features of this biomaterial is the composition and properties of the cells entrapped within the fibrin matrix, as these cells actively contribute to its regenerative potential. Our previous findings described CGF as a heterogeneous structure: the outer portion consists of a dense, compact fibrin network, whereas the inner portion contains a rich, uniformly distributed cell population embedded within the three-dimensional fibrin matrix [12,13].

Gene expression analyses have demonstrated that cells isolated from CGF express pluripotency-associated genes such as Oct3/4 and Nanog. Oct3/4 plays a critical role in maintaining stem cell identity and is a key transcription factor in multipotent progenitors, whereas Nanog is essential for self-renewal in embryonic stem cells [17]. These data highlight that CGF cells exhibit marked plasticity, as it has been previously demonstrated that they can differentiate into osteoblasts [12,13].

Furthermore, CGF has been shown to promote neuronal recovery and regenerative processes [9,10]. In particular, studies using CGF prepared from rat venous blood and applied to nerve injuries demonstrated enhanced Schwann cell migration and β1 integrin activation, ultimately facilitating neuronal regeneration [18]. Recent clinical studies have also shown that combining autologous CGF with adipose tissue grafts improves the survival of transplanted tissue when used to treat facial contour deformities [19]. Additionally, in vitro experiments have revealed that CGF can stimulate the proliferation, migration, and angiogenic differentiation of human umbilical vein endothelial cells (HUVECs) [13], indicating its potential application in revascularization therapies [20].

Although the osteogenic potential of these cells has been well characterized, their ability to differentiate into other lineages remains largely unexplored. Given the multipotent nature of the CGF cells, this study aims to investigate their in vitro behavior in order to assess their potential to differentiate into alternative phenotypes.

To this end, primary cells were isolated from CGF and cultured under different conditions using lineage-specific induction media to promote differentiation into three cell types of clinical and regenerative interest: adipocytes, endothelial cells, and neurons.

The objective of this study is therefore to further elucidate the plasticity of CGF primary cells (CPCs) by exploring their differentiation potential beyond osteogenesis, and to identify novel clinical applications in the field of tissue regeneration.

## 2. Results

### 2.1. CPCs Express Stem Markers

**Figure 1:**
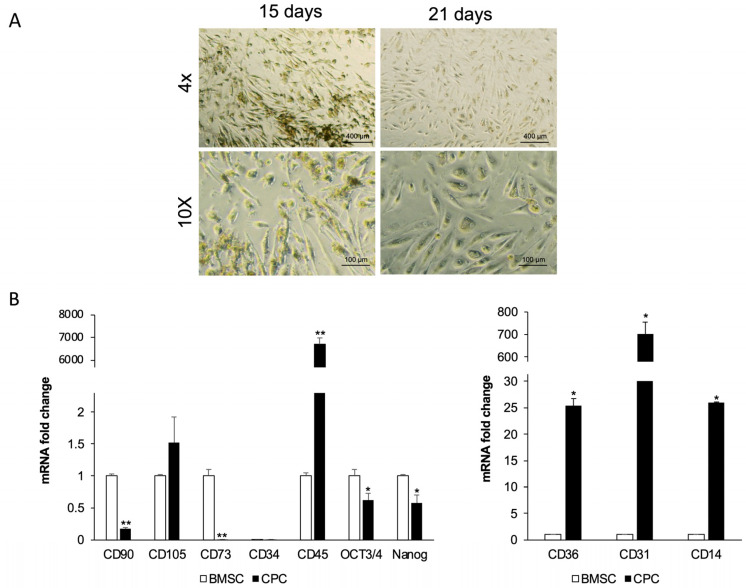
Morphological and immunophenotypic profile of CPCs. (**A**) The micrographs showed CPCs released by CGF pieces after 15 days and 21 days from cutting. They are representative of three independent experiments. (**B**) Real-time PCR analysis of stem cell surface and pluripotent markers in CPCs compared with BMSCs. *Gapdh* was used as a housekeeping gene. The results are expressed as means ± SD of triplicate measurements from four independent experiments. * *p* ≥ 0.05, ** *p* ≥ 0.01 relative to BMSCs.

After 15 days in culture medium, the CGF was cut into fragments and incubated in fresh medium. Cells released from the CGF fragments, after approximately one month in culture, showed morphologically heterogeneous (Figure 1A), including a high percentage of elongated cells that increased over time (21 days). To characterize these cells, we performed quantitative real-time PCR to analyze the expression of mesenchymal stromal cell and hematopoietic stem cell markers, as well as stemness markers, and compared them with those commonly expressed by human bone marrow mesenchymal stem cells (BMSCs).

Quantitative analysis revealed that CPCs expressed levels of CD105 mRNA at levels comparable to BMSCs. However, unlike BMSCs, CPCs did not express typical mesenchymal stem cell markers such as CD90 and CD73 (Figure 1B). Neither cell population, CPCs or BMSCs, expressed the hematopoietic stem cell marker CD34, but the CPCs displayed markedly higher CD45 mRNA levels compared to BMSCs, in which this marker is absent. Although to a lesser extent than BMSCs, CPCs showed detectable OCT3/4 and NANOG mRNA, two transcription factors crucial for maintaining pluripotency.

Furthermore, CPCs showed CD14 and CD36 mRNA levels approximately 26-fold and 25-fold higher, respectively, than those observed in BMSCs, and exhibited high levels of CD31 mRNA.

### 2.2. CPCs Differentiate into Adipocytes 

**Figure 2:**
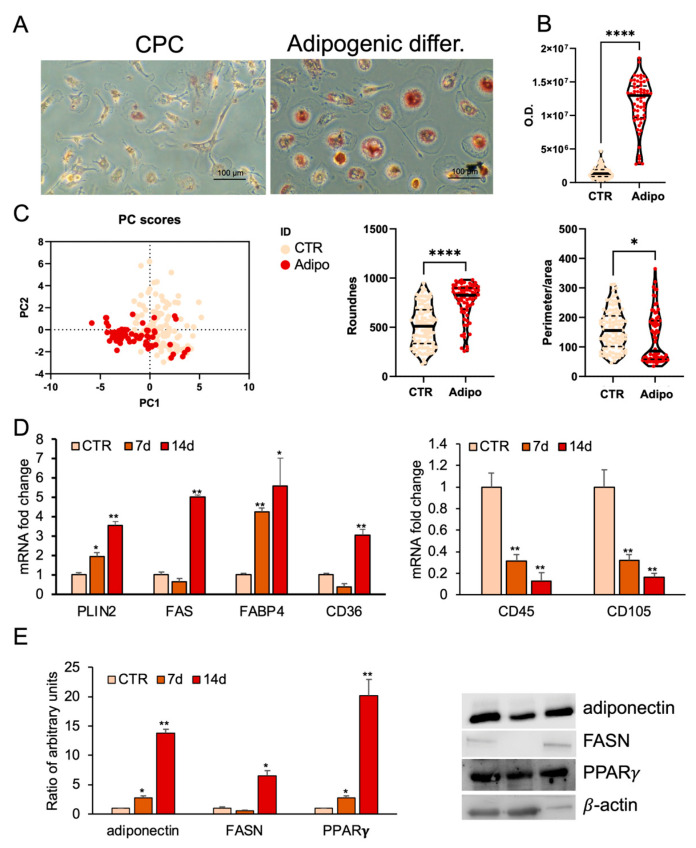
Adipogenic differentiation of CPCs. (**A**) Oil Red O (ORO) staining in CPCs grown in basal medium (CTR) or adipogenic medium within 2 weeks. Images are representative of three independent experiments. (**B**) ORO staining intensity was quantified using image analysis ImageJ software (1.54v). (**C**) Morphometric analysis of roundness and the ratio between perimeter and area of CPCs grown in basal medium (CTR) or in adipogenic medium at 14 days, analyzed using ImageJ software (version 1.54f). (**D**) Real-time PCR analysis of stemness and adipocyte-specific gene expression. Total RNA was extracted from CPCs grown in basal medium (CTR) or in adipogenic medium at days 7 and 14. The mRNA expression of stemness and adipocyte-specific genes was analyzed by real-time PCR and normalized with *Gapdh*. (**E**) Protein expression levels in total protein extracts of CPCs grown in basal medium (CTR) or adipogenic medium at days 7 and 14. Western blots were probed with specific primary antibodies against FASN, adiponectin, and PPARγ. Blots of total extracts were normalized vs. β-actin. Each bar represents the mean ± standard deviation (SD) (*n* = 3). * *p* ≥ 0.05, ** *p* ≥ 0.01, **** *p* < 0.0001 relative to CTR.

To evaluate adipogenic differentiation, lipid droplet formation was analyzed by Oil Red O (ORO) staining after 14 days of adipogenic induction. ORO staining revealed a significant increase in lipid droplet formation in treated CPCs compared with controls. Moreover, CPCs cultured in adipogenic medium changed morphology from spindle-shaped to spherical (Figure 2A). The measurements of absorbance of ORO staining confirmed a marked increase in intracellular lipid accumulation in adipogenic medium-treated cells as compared with controls (Figure 2B).

Principal component analysis (PCA) summarized the main variations in the dataset. CPCs treated with the adipogenic medium clustered separately from controls, suggesting that the treatment induced consistent and specific changes, most likely related to the adipogenic differentiation process (Figure 2C).

Morphometric analysis showed that treated cells had a significantly higher roundness value, reflecting a more spherical shape, and a reduced perimeter-to-area ratio, consistent with a more compact and regular morphology (Figure 2C).

Furthermore, CPCs reduced the expression of CD45 and CD105 surface markers after adipogenic differentiation compared to control cells (Figure 2D).

At 7 and 14 days post-induction, quantitative real-time PCR confirmed time-dependent increases in adipogenic markers. PLIN2 and FABP4 mRNA levels increased progressively, while FASN and CD36 mRNA levels were significantly upregulated at 14 days but not at 7 days compared to undifferentiated cells (Figure 2D). Western blot analysis showed that undifferentiated CPCs weakly expressed PPARγ, adiponectin, and FASN proteins; however, their expression strongly increased after the adipogenic treatment (Figure 2E).

**Figure 3:**
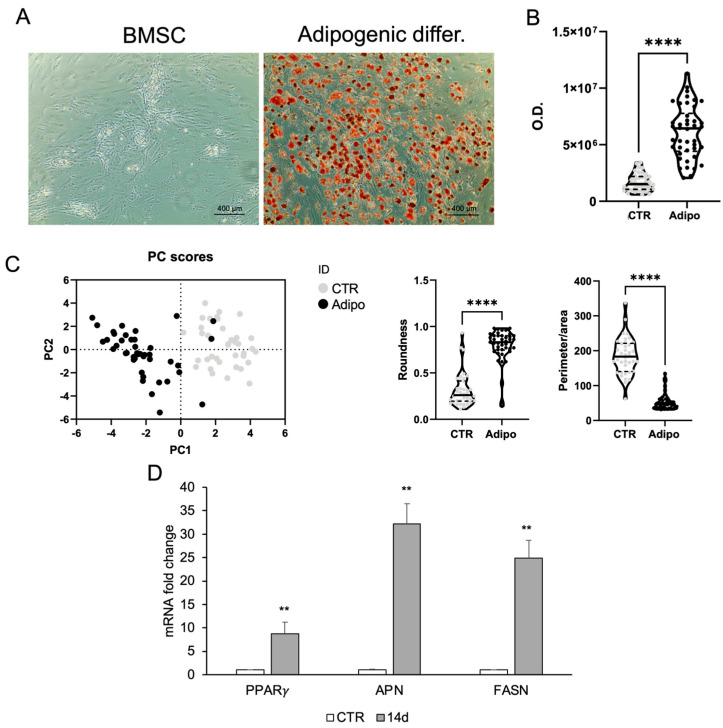
Adipogenic differentiation of BMSCs. (**A**) ORO staining in BMSC growing in basal medium (CTR) or adipogenic medium within 2 weeks. (**B**) ORO staining intensity was quantified using image analysis ImageJ software (1.54v). (**C**) Morphometric analysis of roundness and the ratio between perimeter and area of BMSCs grown in basal medium (CTR) or in adipogenic medium at day 14, analyzed using ImageJ software (version 1.54f). (**D**) Real-time PCR analysis of adipocyte-specific gene expression. Total RNA was extracted from BMSCs grown in basal medium (CTR) or in adipogenic medium at day 14. The mRNA expression of stemness and adipocyte-specific genes was analyzed by real-time PCR and normalized with *Gapdh*. Each bar represents the mean ± SD (*n* = 3). ** *p* ≥ 0.01, **** *p* < 0.0001 relative to CTR.

A commonly used cell line, valued for its plasticity and differentiation capacity, is represented by BMSCs. The ORO staining revealed strong lipid accumulation in BMSCs treated with adipogenic medium for 14 days, whereas control cells showed no staining. Morphological analysis confirmed these changes (Figure 3).

### 2.3. CPCs Differentiate into Endothelial Cells

**Figure 4:**
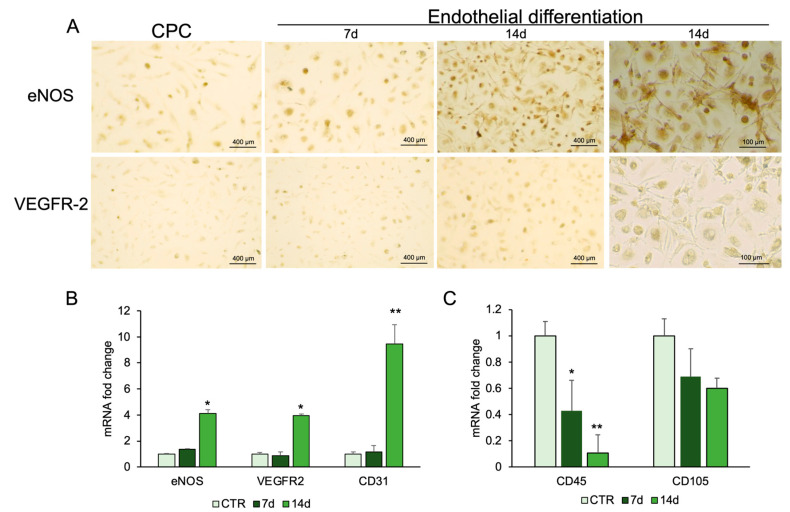
Endothelial differentiation of CPCs. (**A**) Immunostaining analysis was performed for eNOS and VEGFR-2. RNA was extracted from CPCs grown in basal medium (CTR) or in endothelial medium at days 7 and 14. The mRNA expression of endothelial-specific genes (**B**). and stemness genes (**C**) was analyzed by real-time PCR and normalized with *Gapdh*. Each bar represents the mean ± SD (*n* = 3). * *p* ≥ 0.05, ** *p* ≥ 0.01 relative to CTR.

To assess endothelial differentiation, CPCs were cultured in endothelial induction medium for 14 days, and marker expression was analyzed by immunocytochemistry and real-time PCR. Endothelial nitric oxide synthase (eNOS) and vascular endothelial growth receptor-2 (VEGFR-2) were detected by immunostaining, supporting endothelial commitment (Figure 4A). Real-time PCR confirmed increased expression of eNOS and VEGFR-2 compared with controls. CD31, another marker of endothelial cells, showed mRNA levels higher in differentiated cells than in control cells (Figure 4B). The expression of hematopoietic, CD45, and mesenchymal markers, CD105, was also evaluated. CD45 mRNA expression was significantly reduced, while CD105 mRNA did not show a reduction in treated cells with respect to control cells (Figure 4C).

**Figure 5:**
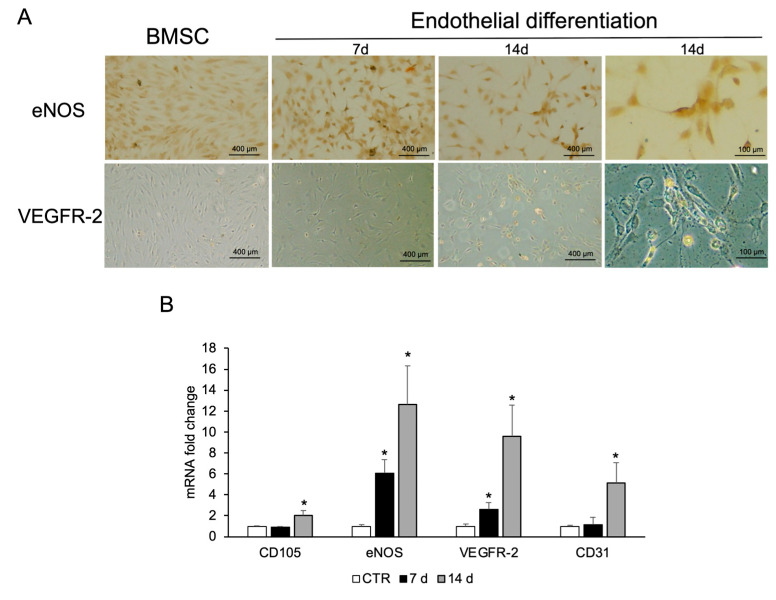
Endothelial differentiation of BMSCs. (**A**) Immunostaining analysis was performed for eNOS and VEGFR-2. Images are representative of three replicates for each condition. (**B**) RNA was extracted from BMSCs grown in basal medium (CTR) or in endothelial medium at days 7 and 14, and the expression of endothelial cell markers (CD105, eNOS, VEGFR-2, and CD31) was analyzed by real-time PCR. *Gapdh* was used as housekeeping gene. Each bar represents the mean ± SD (*n* = 3). * *p* ≥ 0.05 relative to CTR.

BMSCs were treated for 7 and 14 days with the same endothelial differentiation medium used for CPCs. Immunostaining revealed high eNOS expression as early as 6 days. The staining intensity, directly proportional to the protein expression levels, was greater after 14 days. The expression of VEGFR-2 is less pronounced compared to eNOS; however, a positive signal becomes evident after 14 days of treatment with respect to undifferentiated BMSC (Figure 5A). This finding is comparable to that observed in CGF-related cells (Figure 4A). BMSCs treated with VEGF for 7 and 14 days showed mRNA of endothelial markers such as CD105, CD31, eNOS, and VEGFR-2 upregulated with respect to CTR, especially at the end of differentiation time (Figure 5B).

### 2.4. Differentiation of CPCs into Neuron-like Cells

To evaluate the ability of CPCs to acquire a neuron-like phenotype, cells were cultured in neuronal induction medium and analyzed by immunocytochemistry, molecular, and protein analyses.

**Figure 6:**
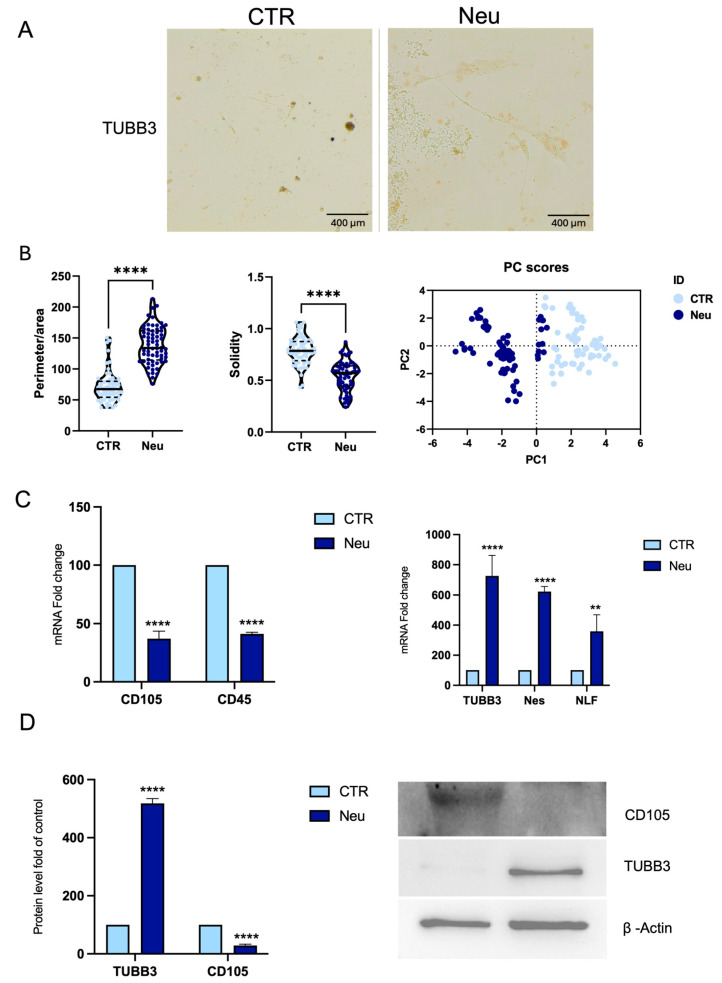
Neuronal differentiation of CPCs. (**A**) Immunohistochemical staining for TUBB3 shows positive labeling in CPCs cultured in neuronal differentiation medium Neu compared to CTR, scale bar 400 μm. (**B**) Morphometric analysis of ratio between perimeter and area, solidity, and PCA. (**C**) RNA was extracted from CPCs, CTR, or in Neu. The mRNA expression of stemness and neuronal-specific genes mRNA abundance was analyzed by real-time PCR. *Gapdh* was used as a housekeeping gene for normalization. (**D**) Western blot and densitometric analysis of TUBB3 and CD105 protein in Neu and CTR cells. β-actin was used as a loading control. Results are expressed as mean ± SD of triplicate measurements (*n* = 3) ** *p* < 0.01, **** *p* < 0.0001 relative to CTR.

As illustrated in Figure 6A, β-Tubulin III (TUBB3) immunohistochemical staining revealed a positive signal in cells treated with the neuronal differentiation medium (Neu), contrasting with the untreated control (CTR). The cells in Neu condition displayed a more extended morphology with thin, elongated cytoplasmic processes consistent with a neuronal-like phenotype. This is supported by the morphological analysis (Figure 6B), which showed an increased perimeter-to-area ratio in the Neu condition, indicating that the cells exhibited a more complex, branched morphology. The significant reduction in solidity in the Neu condition is consistent with enhanced neuronal differentiation, involving the extension of cellular processes and resulting in a less compact shape. Finally, the PCA demonstrates that the observed morphological features effectively discriminate between the two conditions (Figure 6B).

Real-time PCR showed reduced CD45 and CD105 expression in Neu cells (Figure 6C), while TUBB3, Nestin (Nes), and Neurofilament (NFL) were significantly upregulated. Western blot confirmed increased TUBB3 protein expression and reduced CD105 levels, consistent with a loss of mesenchymal features (Figure 6D).

**Figure 7:**
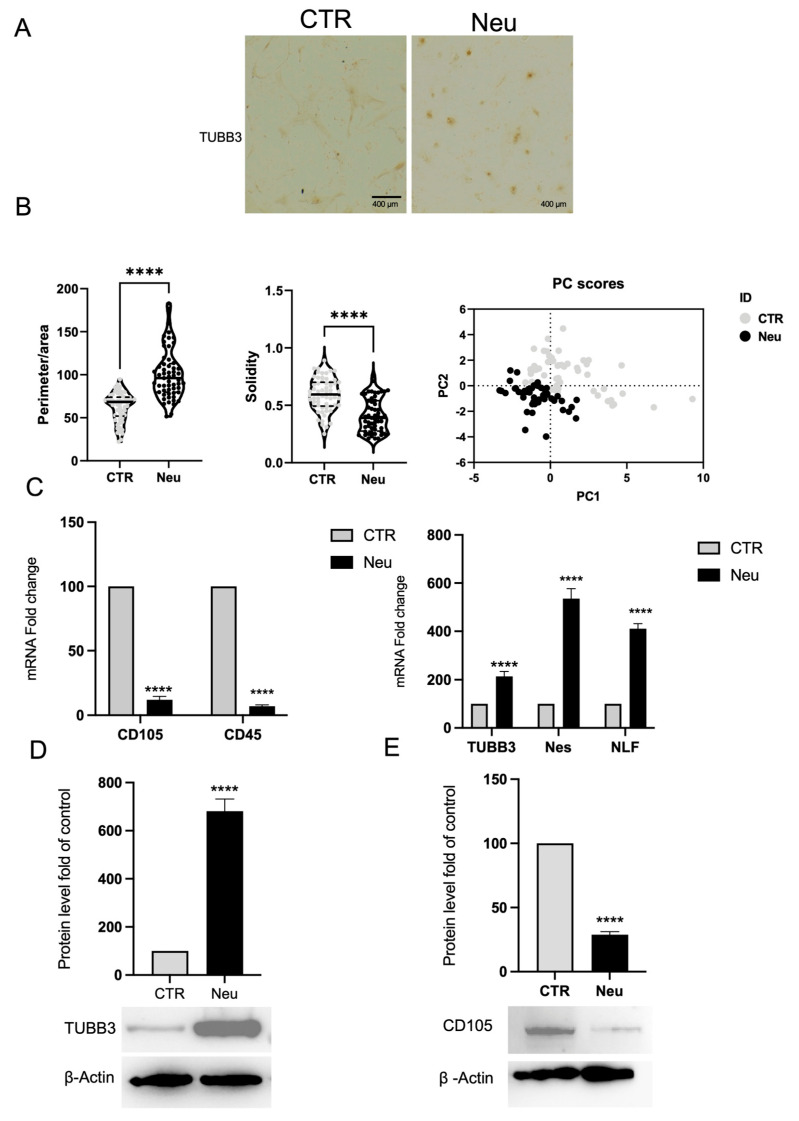
Neuronal differentiation of BMSCs. (**A**) Immunohistochemical staining for TUBB3 shows positive labeling in BMSCs. Cultured in neuronal differentiation medium (Neu) compared to CTR, scale bar 400 μm. (**B**) Morphometric analysis of the ratio between perimeter and area, solidity, and PCA. (**C**) Real-time PCR analysis reveals downregulation of hematopoietic (CD45) and mesenchymal (CD105) markers and upregulation of neuronal genes, including TUBB3, Nes, and NLF, in Neu cells. *Gapdh* was used as a housekeeping gene for normalization. (**D**) Western blot and densitometric analysis confirm increased TUBB3 protein expression in Neu cells compared to CTR, and (**E**) Western blot analysis shows reduced CD105 expression in Neu cells, indicating a loss of mesenchymal traits. β-actin was used as a loading control. Results are expressed as mean ± SD of triplicate measurements from three independent experiments. **** *p* < 0.0001 relative to control.

In parallel with the experiments performed on primary cells released from CGF, a neuronal differentiation treatment was carried out on BMSCs to evaluate the efficiency of converting them to a neuronal fate. As illustrated in Figure 7A, immunocytochemistry for TUBB3 revealed a significant increase in immunoreactivity in cells Neu, contrasting with CTR, which retained a fibroblast-like morphology and exhibited minimal expression of the TUBB3 marker. The morphological change, confirmed by qualitative analysis, is illustrated in the graphs showing the analysis of the perimeter/area ratio, solidity, and PCA (Figure 7B).

The morphological observations were corroborated by gene expression analysis using real-time PCR. Figure 7C illustrates a significant decrease in the mesenchymal markers CD105 and CD45 mRNA abundance in the treated cells compared to the control cells, indicating a loss of the original mesenchymal phenotype. Conversely, a significant upregulation of genes typically associated with the neuronal phenotype, such as Tubb3, Nestin, and NFL, was detected (Figure 7C), confirming the acquisition of a neuronal identity at the transcriptional level.

Protein expression analysis by Western blot confirmed these findings. As illustrated in Figure 7D,E, the treated cells exhibited a significant increase in TUBB3 protein level, consistent with the transcriptional data, and a notable decrease in CD105 protein level. β-actin expression remained constant across samples and was used as an internal loading control, supporting the conclusion that neuronal differentiation was efficiently induced.

## 3. Discussion

CGF has recently gained attention as a versatile tool in regenerative medicine, with applications extending beyond traditional wound healing to encompass aesthetic, reconstructive, and even oncological settings. Its growing interest is largely attributable to its potent regenerative properties and the broad clinical potential associated with its unique composition [21].

Our study aimed to determine whether CGF can be considered a reservoir of autologous stem cells with plasticity comparable to BMSCs, and whether this characteristic, combined with its high content of growth factors, may explain its ability to accelerate the healing of wounds, including chronic lesions, more rapidly than other platelet derivatives [8,22].

In clinical practice, CGF has been shown to promote hair regrowth in androgenetic alopecia, where a single subcutaneous injection of CGF into alopecic areas sustained hair regrowth for up to six months post-application [23]. Promising outcomes have also been reported in anti-aging therapies and plastic surgery, particularly in facial rejuvenation procedures [24]. In reconstructive surgery, CGF has been applied to enhance the repair of both hard and soft tissues. A well-established application is in the dental field, particularly in oral and maxillofacial surgery, including the treatment of temporomandibular disorders [25,26]. A recent meta-analysis, which includes a randomized controlled trial, demonstrates that the coronally advanced flap combined with CGF significantly improves clinical outcomes such as root coverage and gingival thickness when compared to the conventional approach [27]. The regenerative capabilities of CGF have also proven effective in managing post-operative complications associated with cosmetic procedures as well. Notably, CGF has been employed to reverse necrotic skin damage resulting from hyaluronic acid filler injections, a rare but severe complication [28]. These therapeutic effects are largely attributed to the gradual release of multiple growth factors embedded in the CGF fibrin matrix. Our previous work demonstrated a programmed and sequential release profile: VEGF peaked at day 14, TGF-β1 and BMP-2 reached maximal concentrations by day 21, and remained elevated until day 28. Moreover, CGF is a source of MMP-2 and MMP-9, matrix metalloproteinases involved in extracellular matrix remodeling and cell migration, which peak around day 7 [12]. This gradual release, supported by the dense fibrin network, ensures a more controlled and less inflammatory delivery of bioactive molecules.

Mechanistically, CGF has been shown to attract fibroblasts by activating the MEK/ERK signaling pathway, thereby promoting tissue regeneration [29]. In addition, CGF enhances revascularization of the damaged area, contributing to more effective tissue recovery [13,30].

We have previously demonstrated that the sustained release of growth factors by CGF is also attributable to the cells entrapped within the fibrin matrix [12,13]. These cells form a heterogeneous population and exhibit a mixed immunophenotypic profile. The cells within the fibrin clot and released in two weeks from CGF expressed CD34, CD45, and CD105, indicating the presence of a cellular pool comprising hematopoietic stem cells, lymphocytes, monocytes, and endothelial progenitor cells (EPCs) [11,12,13]. The release of EPC-like cells from CGF was confirmed by the expression of CD34 and endothelial markers, including eNOS, VE-cadherin, VEGFR-2, and CD31. These EPC-like cells promote angiogenesis by producing angiogenic factors such as VEGF, TGF-β1, MMP-2, and MMP-9, actively participating in neovascularization and tissue remodeling. They are also able to integrate into pre-existing endothelial tubules, contributing to vascular structure formation in vitro [13].

In the present study, we isolated the cells by cutting the CGF after 15 days of incubation in medium. The CGF pieces released heterogeneous cell populations with a higher percentage of elongated cells compared to those released from intact CGF during the first 15 days [13].

After one month of culture, CGF-derived cells no longer expressed the hematopoietic marker CD34, which was observed in CGF cells at shorter culture times [12,13]. However, they expressed high levels of monocyte-associated markers such as CD31, CD36, CD14, and CD45. Circulating monocytes are known to exhibit considerable plasticity and differentiation capacity under specific growth factor conditions [5,31]. In our experimental conditions, the growth factor-rich environment provided by CGF may be responsible for maintaining the plasticity of these cells. Interestingly, CPCs also expressed stemness markers typically associated with mesenchymal stromal cells, including significant levels of CD105, Oct3/4, and Nanog, comparable to those observed in BMSCs, indicating that their differentiation potential was preserved after long-term in vitro expansion.

However, can these cells differentiate into various cell types in response to different microenvironments and thus actively contribute to the regeneration at the injury site? To address this question, we induced the differentiation of CPCs into adipocytes, endothelial cells, and neuron-like cells.

CPCs cultured in adipogenic medium expressed high mRNA and protein levels of adipocyte-specific markers such as FASN, PPARγ, and CD36. A marked accumulation of intracellular lipid vesicles further confirmed the ability to acquire a functional adipocyte phenotype. Overall, these results clearly and effectively demonstrate that primary cells respond to adipogenic treatment by acquiring a phenotype consistent with that of adipocytes, both morphologically and biochemically. The combination of quantitative, morphological, and statistical analyses strengthens the evidence supporting the success of the differentiation process.

The CPCs treated with endothelial differentiation medium expressed endothelial cell markers such as eNOS and VEGFR-2, as revealed by immunocytochemistry and quantitative real-time PCR. In addition, at 14 days after endothelial induction, CPCs increased CD31 mRNA levels compared to undifferentiated cells. However, a downregulation of stemness marker CD45 mRNA was shown in endothelial differentiated cells, whereas they exhibited high levels of CD105, consistent with its known role in endothelial lineage commitment [32].

The CPCs can differentiate into neuron-like cells following a multistep differentiation protocol. The field of neurology supports the use of autologous formulations with a high platelet content to promote neurogenesis and the recovery and regeneration of neuronal structures [9,10]. Yang et al. evaluated the efficacy and underlying mechanisms of CGF in promoting facial nerve regeneration. Their study resulted in accelerated axonal and myelin repair, as well as increased proliferation of Schwann cells. In vitro, CGF was found to promote the proliferation and migration of RSC96 cells, as well as to facilitate axonal outgrowth in NG108-15 cells [33].

Our results suggest that, in the Neu condition, cells exhibit stronger TUBB3 expression and more complex morphologies. Morphometric metrics (perimeter/area and solidity) confirm a phenotype consistent with neuronal differentiation. PCA analysis further supports the notion that the two populations are phenotypically distinct. This suggests that the treatment induces consistent and reproducible changes in cell morphology, and the quantitative analysis showed the expression of neuronal markers. These findings support the neuroregenerative potential of CPCs.

In addition to adipogenic, endothelial, and neuronal differentiation, we have previously demonstrated that CPCs can differentiate into osteoblasts by progressively downregulating stemness markers and acquiring osteogenic-specific genes such as RUNX2 and osteocalcin under osteoinductive conditions [12]. Osteogenic differentiation was further enhanced by culturing on silicon-doped hydroxyapatite scaffolds [34]. To support our results, multipotent stem cells capable of differentiating into five different cell lineages for regenerative medicine applications have been isolated from platelet derivatives [6,35]. Furthermore, monocytes exhibit features of adult stem cells capable of differentiating into mesenchymal lineages [5], and the CPCs shared characteristics with monocytes.

Future investigations will aim to evaluate the chondrogenic potential of CPCs to provide a more comprehensive understanding of the multipotent characteristics and to expand their translational applications to cartilage repair and osteoarticular regenerative medicine.

A major limitation of this study is the restricted number of donors from whom the cells were isolated. All participants included in the study were healthy individuals, and in our analysis, we did not observe a significant impact of donor sex or age on CPCs’ immunophenotype and differentiation potential. The donor’s physiological condition undoubtedly influences the ability of CGF to contain and release multipotent cells. However, further studies involving patients requiring CGF-based treatments are necessary to assess whether the content and differentiation potential of CPCs vary under pathological or clinically relevant conditions. Moreover, it is important to consider that the number of peripheral blood stem cells increases following tissue injury, as part of a systemic regenerative response triggered by inflammatory and chemotactic signals. This mobilization process facilitates the recruitment of progenitor cells to the damaged site to support tissue repair [36].

In conclusion, this work demonstrates that CGF contains CPCs with stemness characteristics that can be expanded in vitro and differentiated into adipocytes, endothelial cells, and neuronal-like cells in response to appropriate differentiation stimuli. The differentiation capacity of CPCs is comparable to that of BMSCs, indicating that CGF serves as an autologous reservoir of multipotent cells while simultaneously acting as a source of bioactive molecules. These findings highlight the dual therapeutic role of CGF as both a delivery system for growth factors and a stem/progenitor cell niche, underscoring its potential for broad applications in regenerative medicine.

## 4. Materials and Methods

### 4.1. Collection of CGF

Venous blood (8 mL) from 5 healthy, non-smoking adult donors, two females and three males, aged between 27 and 50 years old, was collected and immediately centrifuged by a Medifuge device (Medifuge MF200; Silfradent srl, Forlì, Italy), at 25 °C, using a program with the following characteristics: 30 s acceleration, 2 min 2700 rpm, 4 min 2400 rpm, 4 min 2700 rpm, 3 min 3000 rpm, and 36 s deceleration and stop, to obtain CGF clot, as previously described [12]. Informed consent was obtained from the donors included in this study in accordance with the Declaration of Helsinki. For each set of experiments, CGFs were prepared from the same blood sample of a single donor.

### 4.2. Isolation and Culture of CGF Primary Cells

The CGF clots were washed twice in PBS and incubated in a sterile dish with 2 mL of DMEM low glucose at 37 °C in a humidified atmosphere with 5% CO_2_ for 14 days. After, that, to obtain CPCs, CGF was cut into eight pieces which were seeded onto 6 cm culture plates and incubated in DMEM low glucose complete, containing 10% fetal bovine serum (FBS), 2 mM glutamine, 100 U/mL penicillin, 100 μg/mL streptomycin, and 2 ng/mL FGF-2 (SRP4037, Sigma-Aldrich Merk Life Science S.r.l., Milan, Italy). CPCs adhered to the culture plate and grew until they reached confluence. The medium was replaced weekly (50% volume). After two passages, primary cells were detached using 0.02% EDTA/0.25% trypsin solution and seeded at a density of 1.5 × 10^4^ cells/mL into a 24-well plate for differentiation.

### 4.3. BMSC Culture

The BMSCs (PCS-500-012, ATCC, Rockville, MD, USA) were cultured at 37 °C in a humidified atmosphere containing 95% air and 5% CO_2_. The cells were cultured in low-glucose DMEM (L0101-500, Voden Medical Instruments, Casorezzo, Italy), supplemented with 10% (*v*/*v*) FBS (S181H-500, Voden Medical Instruments), 1% (*v*/*v*) penicillin–streptomycin solution (P4333, Sigma-Aldrich, Milan, Italy) and 2 mM L-glutamine (X0550-100, Voden Medical Instruments), and 2 ng/mL FGF-2. Cultures were reseeded at ~80% confluence, with seeding density adjusted to plate dimensions.

### 4.4. Adipogenic Differentiation

To induce adipogenic differentiation, CPCs or BMSCs were cultured in adipogenic medium (high-glucose DMEM supplemented with 10% FBS, 2 mM glutamine, 100 U/mL penicillin, 100 μg/mL streptomycin, 0.5 mM IBMX (I7018), 1 μM dexamethasone (D4902), 10 μM insulin (I3536), and 200 μM indomethacin (I7378); all from Sigma-Aldrich). Adipogenic differentiation was evaluated by determining the expression of genes involved in adipogenesis, such as PPARγ, PLIN2, FAS, FABP4, CD36, and adiponectin. Lipid droplets were revealed by Oil Red O staining (O1391, Sigma-Aldrich).

### 4.5. Endothelial Differentiation

To induce endothelial differentiation, CPCs or BMSCs were cultured in M199 medium supplemented with 2% FBS, 2 mM glutamine, 100 U/mL penicillin, 100 μg/mL streptomycin, and 50 ng/mL VEGF. Medium was replaced every three days. Endothelial differentiation was evaluated by determining the expression of endothelial markers like eNOS, VEGFR-2, and CD31.

### 4.6. Neuronal Differentiation

At confluence, CPCs and BMSCs cells were divided into two groups, named control (CTR) and neuronal (Neu). In the CTR group, cells were cultured in DMEM low glucose medium with 10% FBS and 2 ng/mL FGF-2. The Neu group followed an 18-day differentiation protocol with four different serum-free DMEM-based media:

(1) 24 h supplemented with 2% B27 (17504-044, Gibco, Segrate, Italy), 250 µM 3-isobutyl-1-methylxanthine (IBMX, 28822-58, Sigma-Aldrich, Merk Life Science S.r.l., Milan, Italy), and 10 ng/mL FGF-2.
(2) 3 days with only 2% B27 supplementation.
(3) 7 days with 1 µM all-trans retinoic acid (sc-200898 Santa Cruz, Segrate, Italy).
(4) 7 days with 2% B27 supplement and 10 ng/mL human brain-derived neurotrophic factor (BDNF, B3795 Sigma-Aldrich).

After 18 days of treatment, the cells were used for subsequent experiments.

### 4.7. Oil Red O Staining

After adipogenic treatment, the CPCs and BMSCs were washed with PBS and incubated with 4% paraformaldehyde in PBS pH 7.4 for 15 min, at room temperature. Then the cells were stained with 5% Oil Red O solution for 30 min in the dark at 37 °C; they were washed three times with PBS, and the lipid droplets were observed and photographed at an inverted microscope.

### 4.8. Immunostaining Analysis

For immunocytochemistry, monolayers of CGF-released cells and BMSCs were prepared in parallel for each differentiation model. They were incubated overnight with antibodies against eNOS at concentrations of 1 μg/mL and with the antibody against VEGFR-2 at a concentration of 0.2 μg/mL, and Tubulin β-III at a concentration of 10 μg/mL. After 3 washes with PBS, the monolayers were incubated for 1 h with a biotinylated anti-mouse IgG antibody (AB_228305 Thermo Fisher, Rodano, Italy) for eNOS and Tubulin β-III or an anti-rabbit IgG antibody (AP132B Millipore) for VEGFR-2. This was followed by a further 1 h incubation with peroxidase-conjugated streptavidin (E2886 Sigma-Aldrich, Merk Life Science S.r.l., Milan, Italy). The monolayers were then incubated with diaminobenzidine (91-95-2 Sigma-Aldrich) for five minutes, washed, and images were acquired at magnifications of 4×, 10×, and 20× using an Invitrogen™ EVOS XL Core imaging system.

### 4.9. Isolation of RNA and Real-Time PCR Analysis

Total RNA was extracted from CGF-released cells or BMSCs seeded in 12-well cultured plates at a density of 1 × 10^4^ for each specific differentiation time. Only RNA without DNA contamination was used for the subsequent preparation of cDNA synthesis. The reverse transcription reaction (20 μL) was carried out using 1 μg of total RNA, 25 ng of random hexamers, and the MultiScribe^®^ Reverse Transcriptase (Applied Biosystem, Monza, Italy), according to the manufacturer’s protocols. Quantitative gene expression analysis was performed in a CFX Connect Real-time System (BioRad, Segrate, Italy) using SsoAdvanced Universal SYBR Green Supermix (BioRad). The quantifications were performed using the ∆∆CT method, and the Glyceraldehyde 3-phosphate dehydrogenase (*Gapdh*) was used as an internal control for normalization [12]. The specificity of the PCR products was confirmed by the melting curve analysis. The sequences of primers used in real-time PCR are reported in Table 1.

### 4.10. Western Blot Analysis

CPCs and BMSCs were seeded under each experimental condition to obtain cellular protein extracts. An equal amount of proteins was loaded onto a 15% polyacrylamide gel, and Western blot was carried out as reported in [37]. Separated proteins were then transferred electrophoretically onto a nitrocellulose membrane (Pall, East Hills, NY, USA). Equal protein loading was confirmed by Ponceau S staining. The filter was blocked with 5% (w/v) non-fat dried milk in buffered saline. Blots were incubated with primary antibodies directed against PPARγ (sc-7273, Santa Cruz, CA, USA), adiponectin (AB3269P, Millipore), FASN (BD-610963, BD bioscience, Milan, Italy), CD105 (sc-18893), Tubulin β-III (sc-80005) or or β-actin (sc-47778), with the latter used as a control. The membranes were incubated with HRP-conjugated secondary antibodies, anti-mouse (A90-116P) and anti-rabbit (A120-101P), for 1 h at room temperature, and immunoreactive bands were identified employing an improved chemiluminescence detection kit (#1705061, Amersham International, Corston Bath, UK). Densitometric analysis was carried out on the Western blots using the ChemiDoc MP Image System (BioRad, Segrate, MI, Italy).

### 4.11. Morphometric Analysis

For morphological assessment, images of cells from each experimental condition were analyzed using ImageJ software (version 1.54f). Cell circularity was calculated using the formula 4π(area/perimeter^2^), roundness was defined as (4 × area)/(π × major axis^2^), and the perimeter-to-area ratio was also considered. Solidity was calculated as the ratio between the cell area and the area of its convex hull. In addition to these parameters, a PCA was performed using a broader set of morphometric descriptors extracted with ImageJ. For each experimental group, between 60 and 80 individual cells were analyzed.

### 4.12. Statistical Analysis

All experiments were independently repeated at least three times. Data are presented as mean values ± SD. Statistical evaluations were performed using GraphPad Prism version 9.5 (GraphPad Software, Boston, MA, USA). Differences between groups were assessed using the Student’s t-test and one-way analysis of variance (ANOVA) followed by the Bonferroni/Dunn post hoc test. Principal component analysis (PCA) was performed on morphometric parameters obtained using ImageJ software. Prior to analysis, variables were normalized to account for differences in measurement scales. PCA was conducted using GraphPad Prism version 9.1 (GraphPad Software, San Diego, CA, USA), and the first two principal components (PC1 and PC2) were extracted to visualize potential clustering among samples. Separate PCA analyses were carried out for different experimental datasets. In Figure 2, PC1 and PC2 explained 40.52% and 20.21% of the variance, respectively. In Figure 3, PC1 and PC2 explained 43.56% and 20.08%, respectively. In Figure 6, PC1 and PC2 explained 40.64% and 16.43%, respectively. In Figure 7, PC1 and PC2 explained 30.38% and 17.40%, respectively.

## Figures and Tables

**Table 1:** Oligonucleotides used for real-time PCR analysis.

Gene	Sequences (5′-3′)	Accession Number
GAPDH	F: ATGGCCTTCCGTGTCCCCAC R: ACGCCTGCTTCACCACCTTC	NM_014364.5
PTPRC (CD45)	F: ATGACCATGTATTTGTGGCTTA R: TGGGGGAAGGTGTTGGGC	NM_080921.3
Endoglin (CD105)	F: TGGGGGAAGGTGTTGGGC R: GCCAGCATTGTCTCACTTCA	NM_001278138.1
Thy1 (CD90)	F: CCACTCTGGCCATTCCC R: GAGCAGGAGCAGCAGCAG	NM_006288.5
CD73	F: AGCTTACGATTTTGCACACC R: CGGATCTGCTGAACCTTGG	BC015940.1
CD34	F: CAATGAGGCCACAACAAACA R: GTGACTGGACAGAAGAGTTT	M81104.1
CD31	F: TGATGCCCAGTTTGAGGTC R: ACGTCTTCAGTGGGGTTGTC	NM_000442.5
CD36	F: ATGCAGCCTCATTTCCACC R: AGGCCTTGGATGGAAGAAC	NM_000072.3
CD14	F: CTGCAACTTCTCCGAACCTC R:CCAGTAGCTGAGCAGGAACC	M86511.1
Oct3	F: TATTCAGCCAAACGACCATC R: GCAGGAACAAATTCTCCAGG	NM_002701.5
Nanog	F: AGATGCCTCACACGGAGAC R: TCTTCTGTTTCTTGACCGGG	NM_024865.2
PLIN2	F: ATGGCAGGCGACATCTACTC R: AAGGGACCTACCAGCCAGTT	NM_001122.4
FASN	F: CCTGCGTGGCCTTTGAAAT R: CATGTCCGTGAACTGCTGC	NM_004104
FABP4	F: GTGGAAGTGACGCCTTTCAT R: TACTGGGCCAGGAATTTGAC	NM_001442.3
APN	F: AGTCTCACATCTGGTTGGGG R: CTCTCTGTGCCTCTGGTTCC	NM_001177800.1
VEGFR-2	F: AGCGATGGCCTCTTCTGTAA R: ACACGACTCCATGTTGGTCA	NM_002253.2
eNOS	F: ACCCTCACCGCTACAACATC R: GCTCATTCTCCAGGTGCTTC	NM_000603.4
Neurofilament	F: CCAAGACCTCCTCAACGTGAAG R: ATGCTTCCCACGCTGGTGAAAC	NM_006158
Nestin	F: GCGGGCTACTGAAAAGTTCC R: CTCCAGGCTGAGGGACATCT	NM_006617.2
Tubulin β-III	F: TCAGCGTCTACTACAACGAGGC R: GCCTGAAGAGATGTCCAAAGGC	NM_006086

## Data Availability

The data presented in this study are available on request from the corresponding author.
